# Supplementary material for: Thermal state and evolving geodynamic regimes of the Meso- to Neoarchean North China Craton
Source: Nat Commun. 2021 Jun 23;12:3888. doi: 10.1038/s41467-021-24139-z (PMC8222299; doi:10.1038/s41467-021-24139-z)
Supplement: Supplementary file 1 — Supplementary Information [file 41467_2021_24139_MOESM1_ESM.pdf]

Supplementary Information for

Thermal state and evolving geodynamic regimes of the Meso- to Neoarchean North China Craton

Guozheng Sun[1], Shuwen Liu[1, *], Peter A. Cawood[2, *], Ming Tang[1], Jeroen van Hunen[3], Lei Gao[1], Yalu Hu[1], Fangyang Hu[4,5,6]

[1]*Key Laboratory of Orogenic Belts and Crustal Evolution, Ministry of Education, School of Earth and Space Sciences, Peking University, Beijing 100871, PR China*

[2]*School of Earth, Atmosphere and Environment, Monash University, Melbourne, VIC 3800, Australia*

[3]*Department of Earth Sciences, Durham University, Durham DH1 3LE, UK*

[4]*Key Laboratory of Mineral Resources, Institute of Geology and Geophysics, Chinese Academy of Sciences, Beijing 100029, China*

[5]*Innovation Academy for Earth Science, Chinese Academy of Sciences, Beijing 100029, China*

[6]*Department of Geosciences, University of Arizona, Tucson, AZ 85721, USA*

Manuscript for *Nature communications*

***Corresponding authors**

Shuwen Liu (swliu@pku.edu.cn)

Peter A. Cawood (peter.cawood@monash.edu)

Supplementary Text

Geological background

The North China Craton (NCC) is one of the largest cratons in eastern Eurasia, with an area of about 300, 000 km^2 (Supplementary Figure 1A), and is composed of several Archean to Paleoproterozoic crystalline basement blocks covered by younger unmetamorphosed Mesoproterozoic to late Mesozoic strata. Previous studies revealed that the NCC experienced a long and complicated geological evolution from 3.8 to 2.5 Ga[1]. Unlike most other ancient cratons in the world, the NCC is characterized by the strong tectono-magmatic activity at ~2.5 Ga, which is an important window to study the crustal growth and evolution and to explore the Archean thermal state and geodynamic regime[2]. The Precambrian metamorphic basement of the NCC is generally considered to be amalgamated by both the Eastern Block (EB) and the Western Block (WB) along the Trans-North China Orogen (TNCO) at ~1.85 Ga (Supplementary Figure 1B)[3].

Tonalite-trondhjemite-granodiorite (TTG) gneisses are the dominant geological signature in the Archean basement blocks of NCC. Supplementary Figure 1B shows the general spatial and temporal distribution of Meso- to Neoarchean TTG gneisses, and detailed zircon age data and sampling locations are summarized in Supplementary Data 3. The magmatic crystallization ages of TTG gneisses are almost continuous from 3.0 to 2.5 Ga, with three peaks occurring at ~2.91, ~2.72 and ~2.53 Ga, and reaching the maximum peak at ~2.53 Ga (Supplementary Figure 1C). The ~2.9 Ga TTG gneisses are sporadically distributed in the Jiaodong, Huoqiu and Eastern Hebei terranes, and ~2.7

Ga ages TTG gneisses exhibit best developed in the North Liaoning-South Jilin, Jiaodong, West Shandong, Dengfeng-Taihua, Zanhuang and Fuping terranes, whereas the ~2.5 Ga data are widespread across the entire EB of NCC (Supplementary Figure 1B). The geological background of each metamorphic terrane is summarized below.

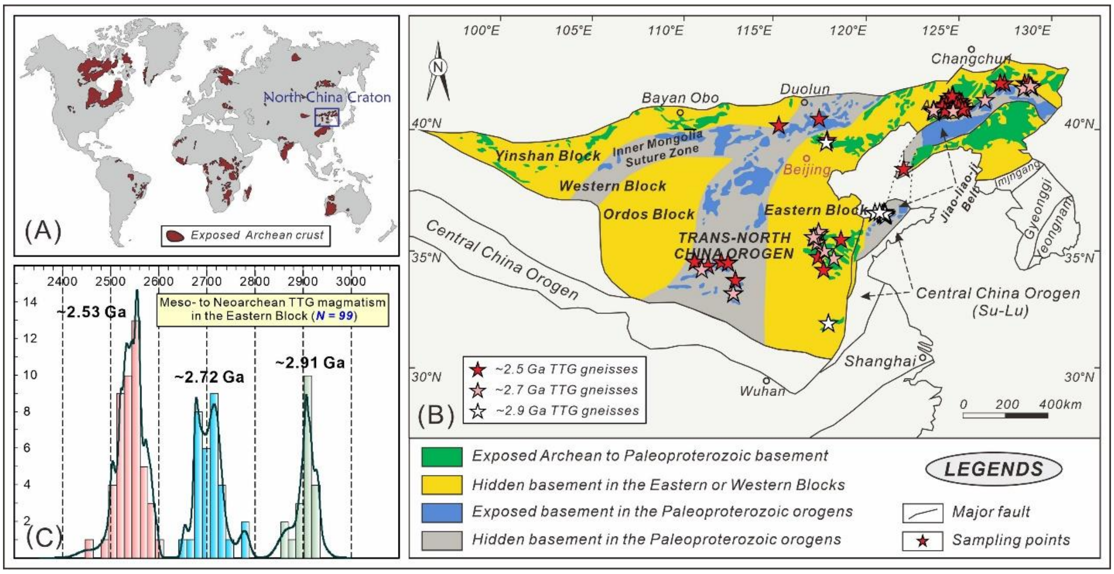

Supplementary Figure 1. (A) The location of the North China Craton in the simplified sketch world map. (B) Tectonic subdivision of the NCC[3], the star showing distribution for the Meso- to Neoarchean TTG gneisses. (C) Age histogram for Meso- to Neoarchean TTG magmatism in the Eastern Block of NCC.

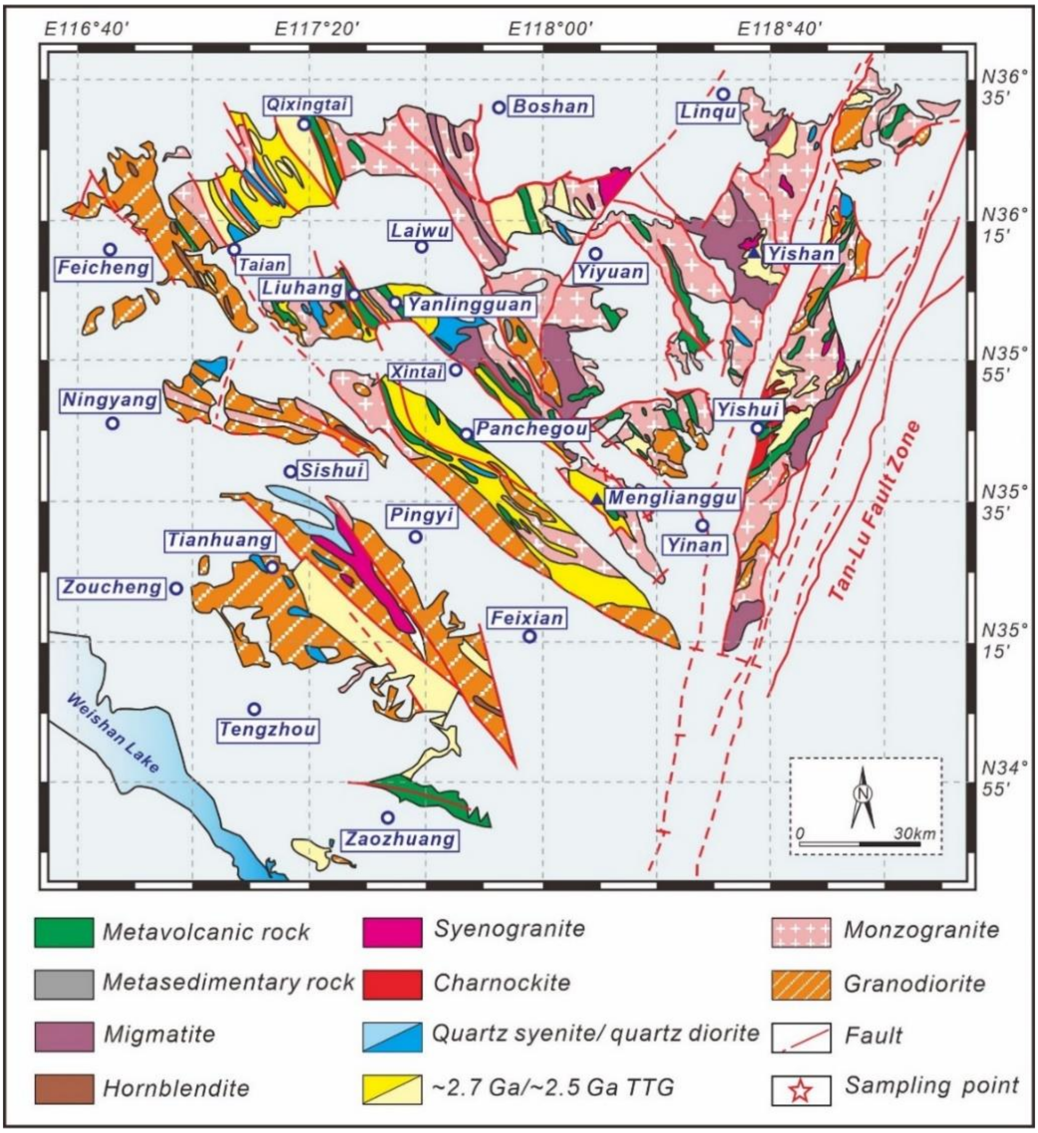

Supplementary Figure 2. Detailed geological map of West Shandong province[4].

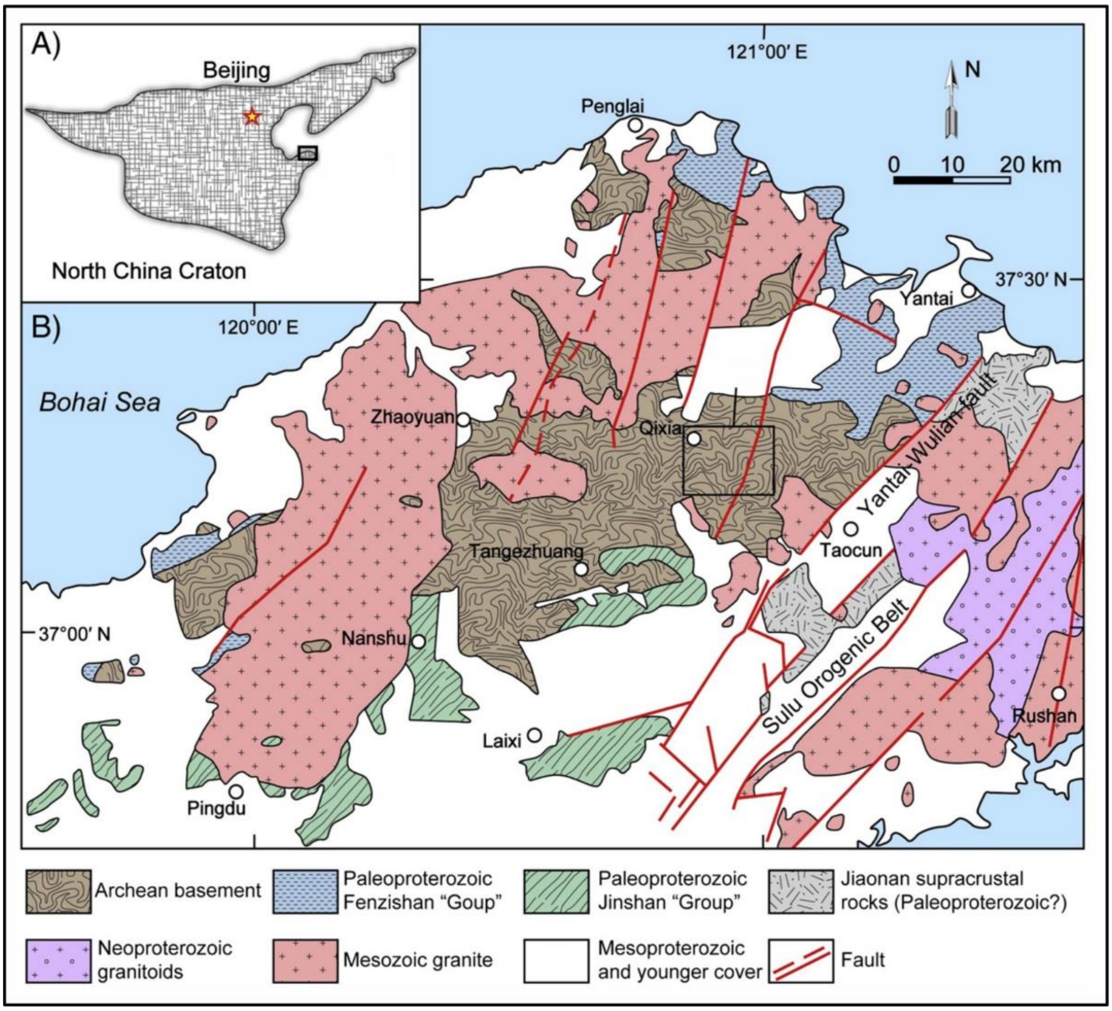

Supplementary Figure 3. (A) Contour of the NCC and locality of Jiaodong terrane. (B) The detailed geological map of Jiaodong terrane[5].

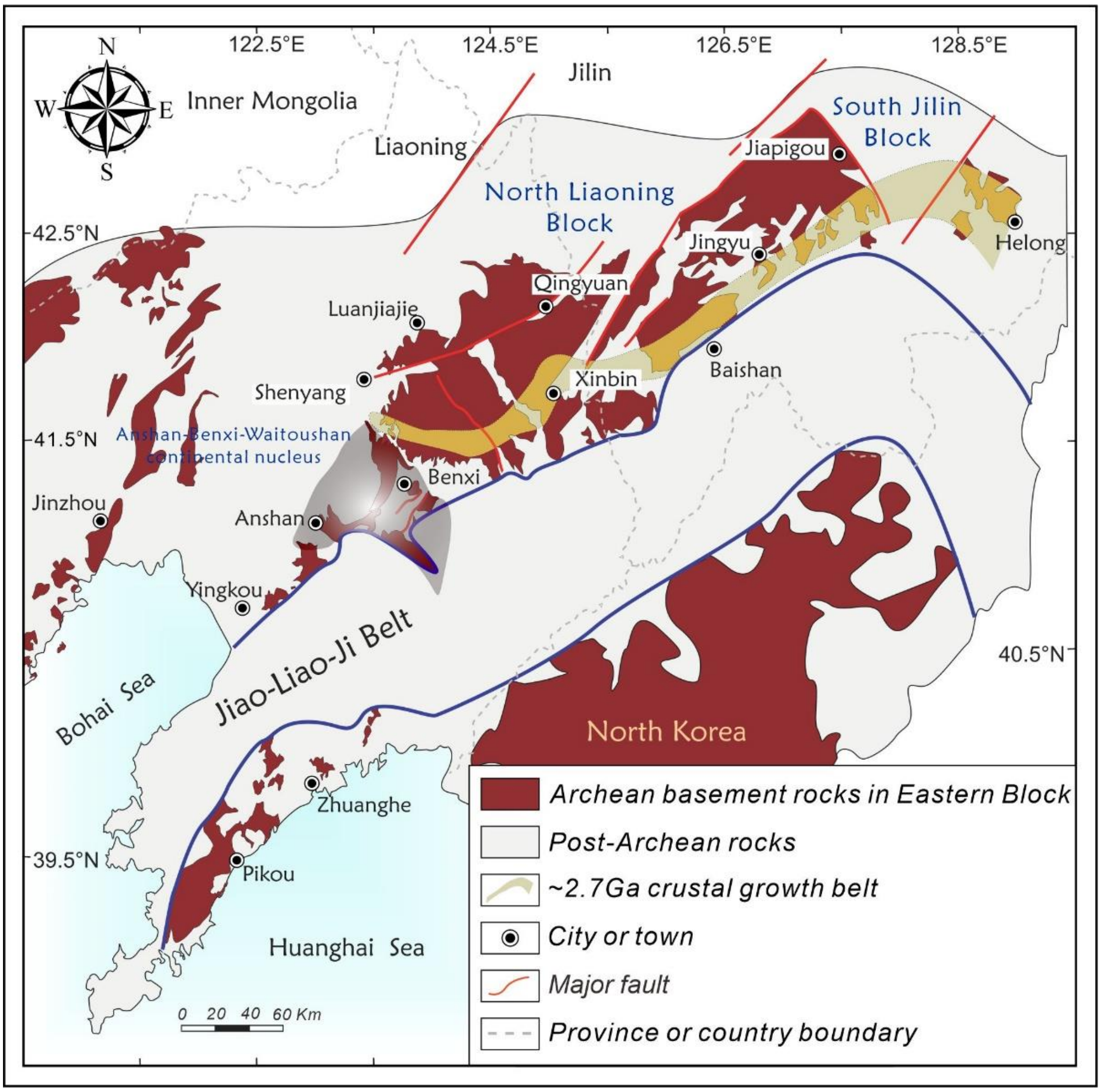

Supplementary Figure 4. Geologic map of the northeastern part of the NCC illustrating major early Precambrian basement terranes and Paleoproterozoic tectonic framework[6,7].

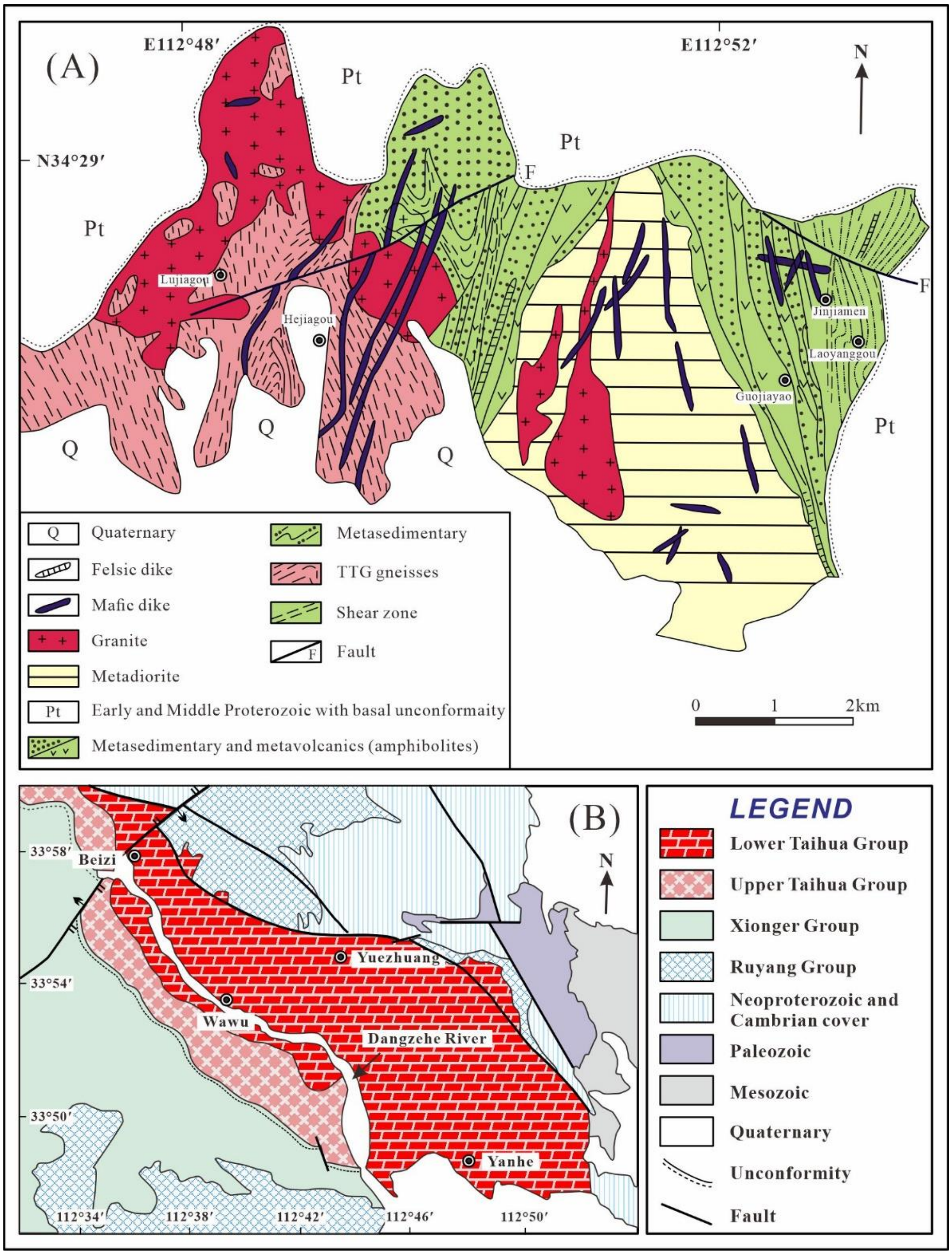

Supplementary Figure 5. (A) Sketch geological map of the Dengfeng complex in the Junzhao area in the southern NCC[8]. (B) Geological map of the Lushan region, illustrating the distribution of the Taihua group[9].

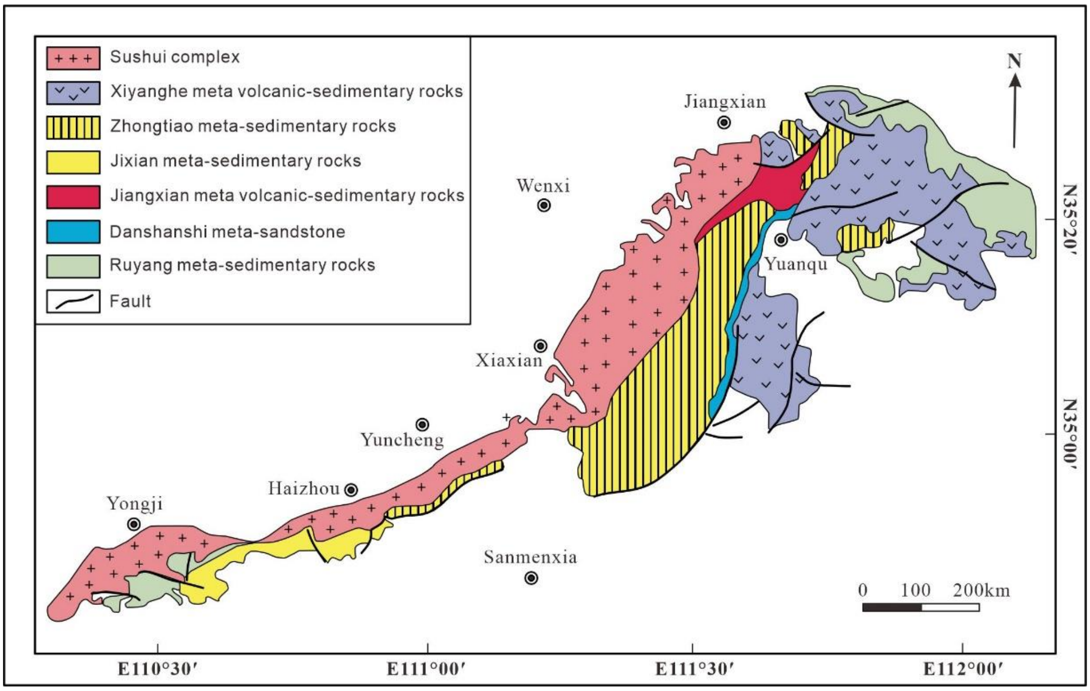

Supplementary Figure 6. Geological sketch map of the Zhongtiao Mountain[10].

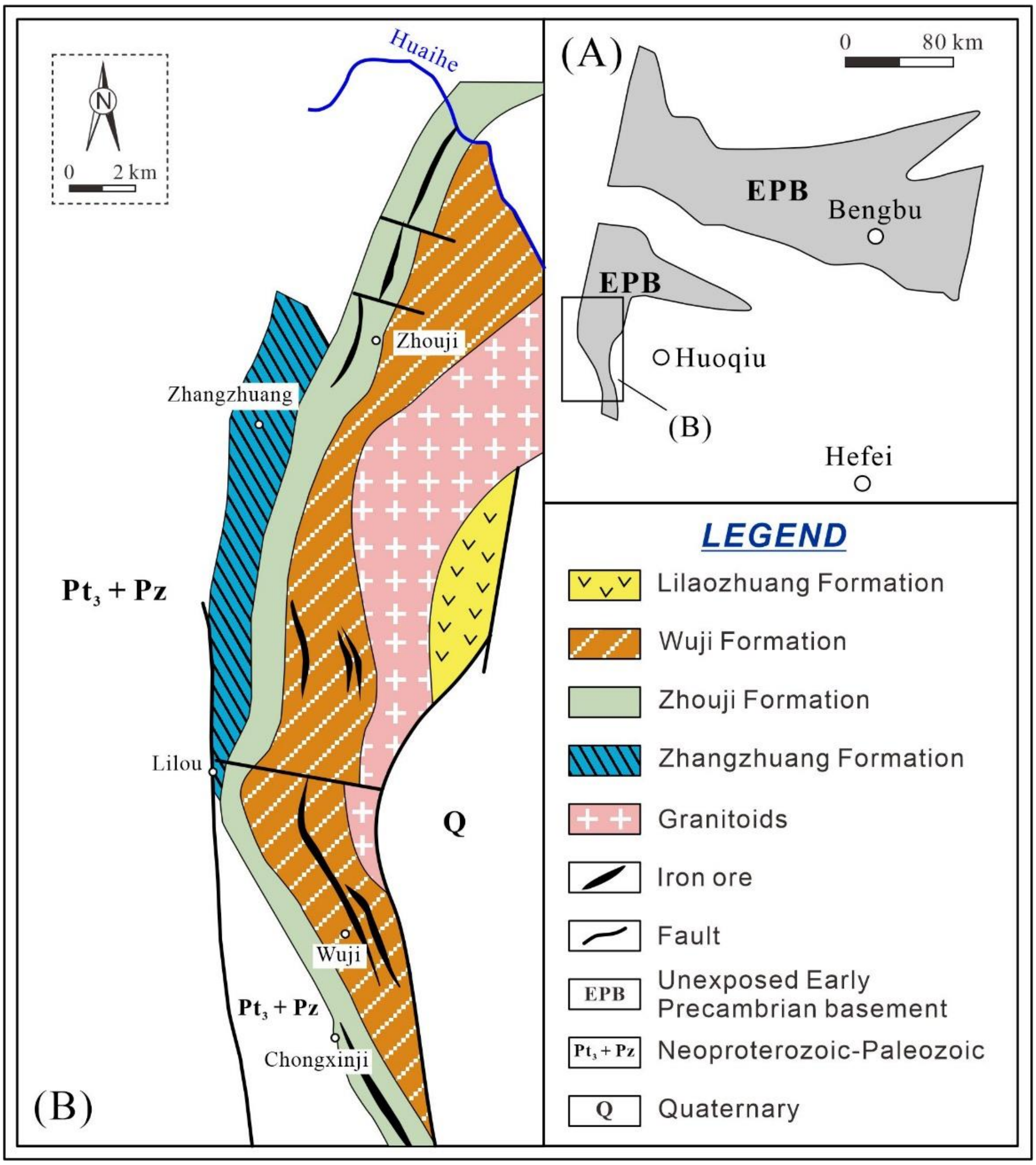

Supplementary Figure 7. (A) The sketch map of Precambrian rocks in the Huoqiu-Bengbu area; (B) Geological map of early Precambrian basement in the Huoqiu area[11].

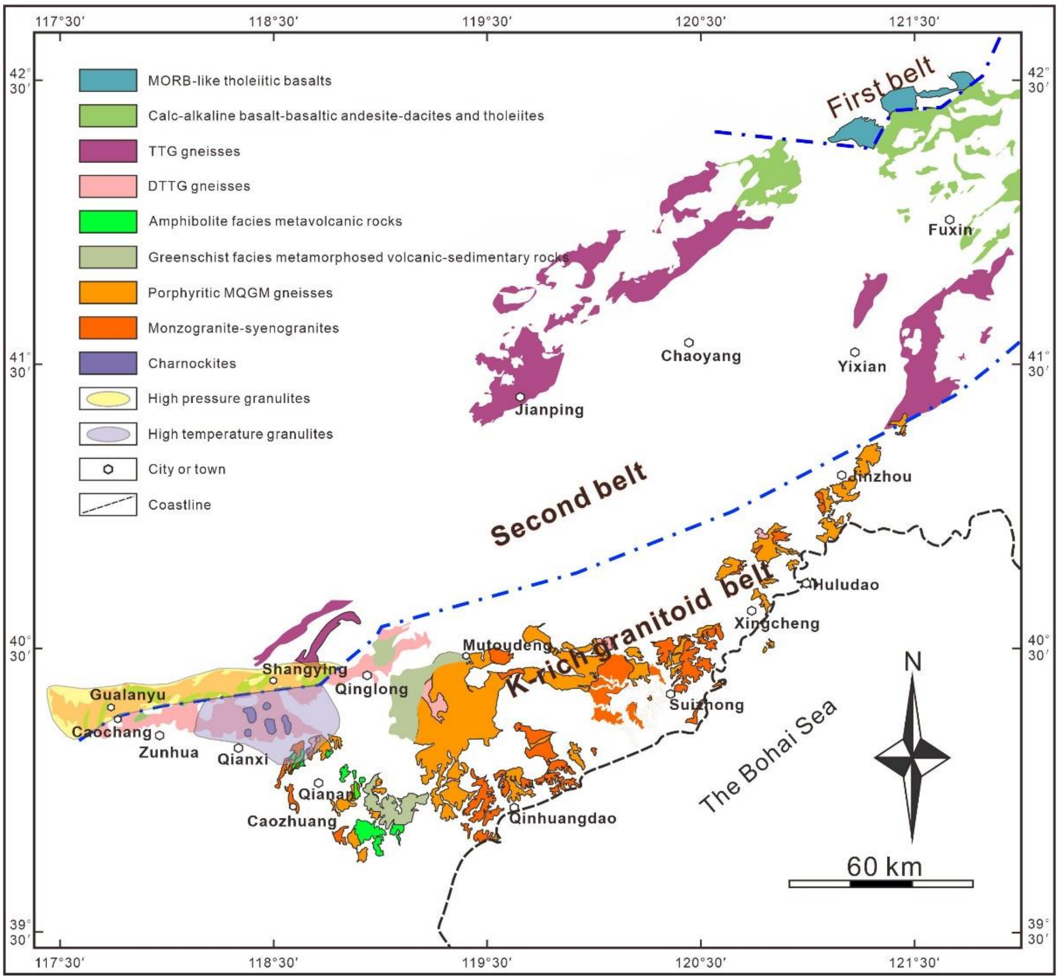

Supplementary Figure 8. Simplified geological map and lithological zoning of the metamorphic basement in the East Hebei-West Liaoning Province[12].

Analytical procedures

Whole-rock samples were firstly trimmed to remove the weathered surfaces, and then were chipped and powdered in an agate mill to about 200 mesh. Major elements were analyzed using X-ray Fluorescence (XRF, Thermal Arl Advant XP+) at the Key Laboratory of Orogenic Belts and Crustal Evolution, Ministry of Education, School of Earth and Space Sciences, Peking University. Loss on ignition (LOI) values were determined by measuring the weight loss after heating the powders at 1050 °C for 30 mins. Sample powders were mixed with lithium tetraborate, and then transformed into

glassy disk by fusion at 1100 °C in a 34-diameter platinum-alloy crucible. The disks were used for major element analyses by XRF. Standards GSR-9 (diorite) and GSR-14 (granitoid gneiss) were used for calibration. The analytical precision is 0.5% for major element oxides.

Sample powders for trace element analyses were pre-treated at Peking University. Firstly, the powders were in turn weighted (25 mg) into Savillex Teflon beakers, placed in a high-pressure bomb with a 1:1 mixture of HF-HNO_3, heated for 24 hours at 80 °C, and then evaporated. After evaporation, 1.5 ml HNO_3, 1.5 ml HF and 0.5 ml $HClO_4$ were added, and the beakers were capped for digestion within a high-temperature oven at 180 °C for 48 hours or longer until the powders were completely digested. Finally, the residue was diluted to 50 ml using 1% HNO_3. Trace elements, including rare earth elements, were measured using an ELEMENT-I plasma mass spectrometer (Finnigan-MAT Ltd.) at the Key Laboratory of Orogenic Belts and Crustal Evolution. The standards GSR-9 (diorite), and GSR-14 (granitoid gneiss) were used for analytical control. The measurement precision of trace elements is better than 5%.

Zircon grains were separated by standard density and magnetic techniques, and then purified by hand picking under a binocular microscope. These zircon grains were mounted in epoxy resin discs, and polished to half the grain thickness. Prior to analysis, cathodoluminescence (CL) images were obtained using a scanning electron microscope at the SEM Laboratory of Peking University. Then, they were analyzed for U–Pb isotopes and trace elements using Agilent 7500 quadruple-based inductively coupled plasma mass spectrometer (ICP–MS) equipped with a GeoLas 193 nm laser (MicroLas,

Göttingen) at the Key Laboratory of Orogenic Belts and Crustal Evolution. The laser beam is 36 μm and frequency is 10 Hz. Harvard zircon 91500 was used as an external standard for zircon U–Th–Pb analyses, and NIST610 as an external standard to calculate the contents of U, Th, Pb, and other trace elements. The $^{207}Pb/^{206}Pb$ and $^{206}Pb/^{238}U$ ratios were calculated using the GLITTER program[13], and common Pb was corrected using the method of Anderson[14]. Age calculations and concordia plotting were carried out using Isoplot (ver. 3.0)[15]. Details of the analytical techniques are documented by Yuan et al.[16].

Zircon Lu–Hf isotopic analyses were conducted on the same or adjacent internal domains as the original pit used for U–Pb isotopic analyses, using a Neptune Plus MC–ICPMS (Thermal Fisher Scientific, Germany) attached to a Geolas 2005 excimer ArF laser ablation system (Lambda physik, Göttingen, Germany) at the state Key Laboratory of Geological Processes and Mineral Resources, China University of Geosciences in Wuhan. Beam diameter of 44 μm and repetition rate of 6 Hz were applied, and zircon 91500 and GJ-1 were used as the external standard and the unknown, respectively. During analysis, every eighth analysis of an unknown was followed by analyses of 91500 and GJ-1. Details of the analytical techniques are documented by Hu et al.[17].

Zircon U–Pb ages of individual samples

Six samples of the Archean TTG gneisses from the Eastern Block were dated by *in-situ* zircon U–Pb methods using LA–ICP–MS at the Key Laboratory of Orogenic Belts

and Crustal Evolution, Ministry of Education, School of Earth and Space Sciences, Peking University. Cathodoluminescence images reveal that some zircon grains exhibit oscillatory or banded zoning, sometimes surrounded by thin dark rims, whereas others are blurred zoned or structureless domains with either bright or dark color. Twenty-five analyses were conducted on samples 18AHB01-1 (granodioritic gneiss), 18AHB02-3 (trondhjemitic gneiss), 18LS02-1 (tonalitic gneiss), 18LS02-2 (tonalitic gneiss) and 18ZT01-1 (tonalitic gneiss), and twenty-six analyses for sample 18AHB02-1 (granodioritic gneiss). Most analyses have Th/U ratios of 0.11–8.60, though some yield lower values of 0.08–0.10. These features indicate that most zircons are of magmatic origin and underwent varying degrees of recrystallization during later tectonothermal events. Analyses of samples 18AHB01-1, 18AHB02-1, 18AHB02-3 and 18ZT01-1 yield four discordant lines, with upper intercept ages of 2911 ± 7, 2930 ± 6, 2931 ± 5 and 2703 ± 6 Ma, respectively (MSWD = 0.47, 0.94, 1.5 and 0.41, Supplementary Figure 9A, B, C and F). These analyses give weighted average mean ages of 2911 ± 10 Ma for 18AHB01-1 (MSWD = 0.16, n = 25), 2934 ± 8 Ma for 18AHB02-1 (MSWD = 0.26, n = 18), 2930 ± 7 Ma for 18AHB02-3 (MSWD = 0.43, n = 20) and 2703 ± 6 Ma for 18ZT01-1 (MSWD = 0.21, n = 25). For samples 18LS02-1 and 18LS02-2, most analyses fall on the concordia lines and yield two concordia ages of 2772 ± 7 Ma (MSWD = 0.0071, n = 19) and 2780 ± 5 Ma (MSWD = 0.022, n = 18) (Supplementary Figure 9D and E). In summary, the TTG gneisses were emplaced during 2703 ± 6 to 2934 ± 8 Ma and subjected to variable degrees of Pb loss.

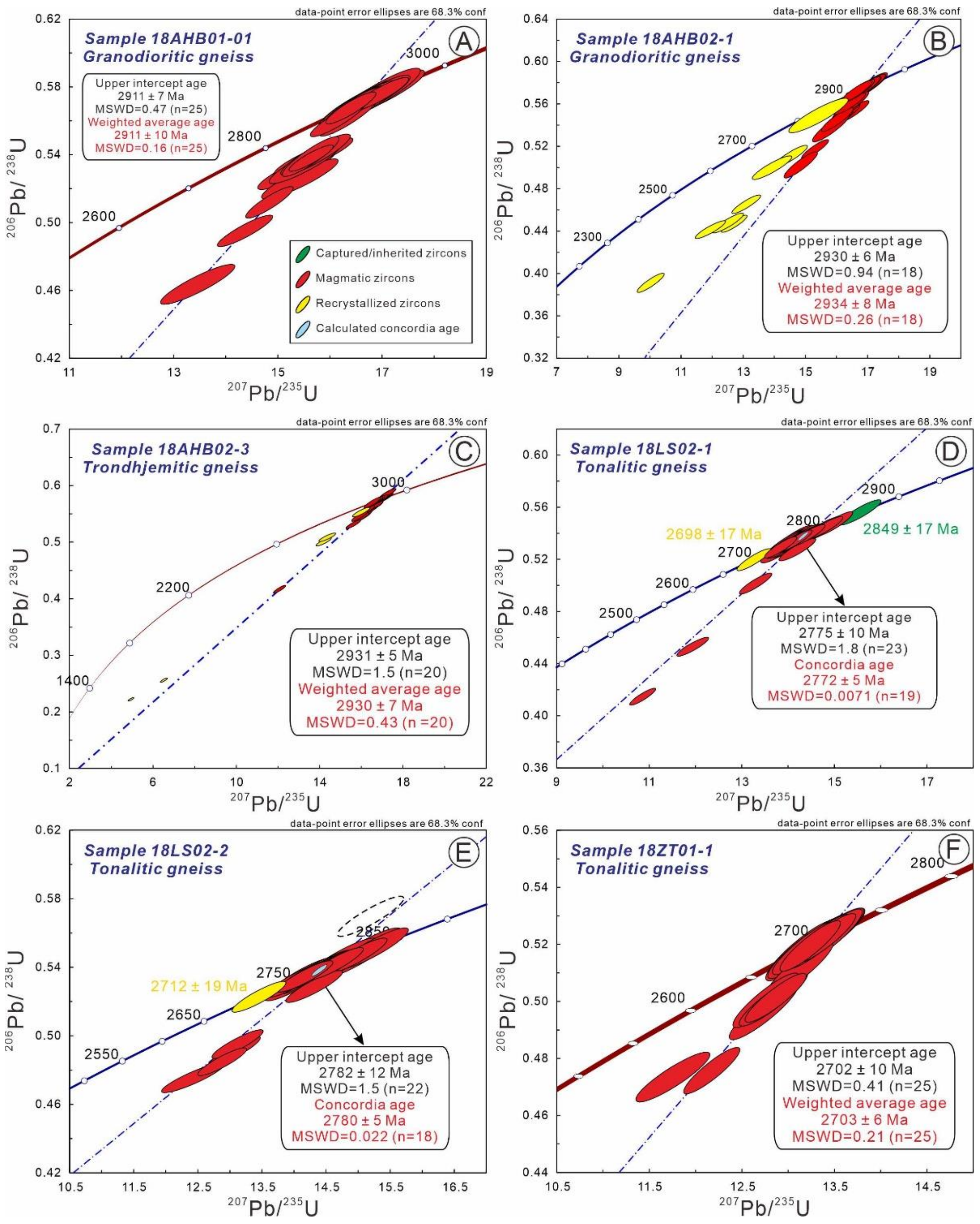

Supplementary Figure 9. Zircon U–Pb concordia diagrams for representative TTG gneiss samples. (A) Granodioritic gneiss (sample 18AHB01-1); (B) granodioritic gneiss (sample 18AHB02-1); (C) trondhjemitic gneiss (sample 18AHB02-3); (D) tonalitic gneiss (sample 18LS02-1); (E) tonalitic gneiss (sample 18LS02-2); (F) tonalitic gneiss (sample 18ZT01-1). Data point error ellipses are 2σ.

Effects of metamorphic and alteration on geochemical compositions

The above petrologic data on the Archean TTG gneisses with different ages in the EB of NCC reveal that these granitoids experienced multiple deformation and metamorphism. Therefore, it is crucial to evaluate the effects of post-magmatic alteration and metamorphism on whole-rock chemical compositions. The studied samples are fresh and the (loss on ignition) values are relatively low (0.16–3.03 wt.%), less than those of altered samples (mostly > 6.0 wt.%)[18]. The uniform chondrite-normalized REE patterns and the absence of evident Ce anomaly (Ce_N/sqrt($La_N \times Pr_N$) of 0.77–1.31) indicate that the original chemical features of these granitoids have not been significantly modified during later metamorphism and alteration[19]. In general, Zr is viewed as one of the most immobile element, which can be applied to assess the mobility of other trace elements. Supplementary Figure 10 shows that La, Sm, Yb, Nb, Ta, Hf, Rb and Sr show good correlations with Zr for the analyzed samples, indicating that these elements were essentially immobile. On the contrary, most samples display poor correlations for Ba, Th and U with Zr, suggesting that these three elements were possibly mobilized. Accordingly, we propose that most TTG gneiss samples preserved the primary geochemical compositions with respect to REE, HFSE, Rb and Sr, whereas Ba, Th and U were modified during the later metamorphic event(s).

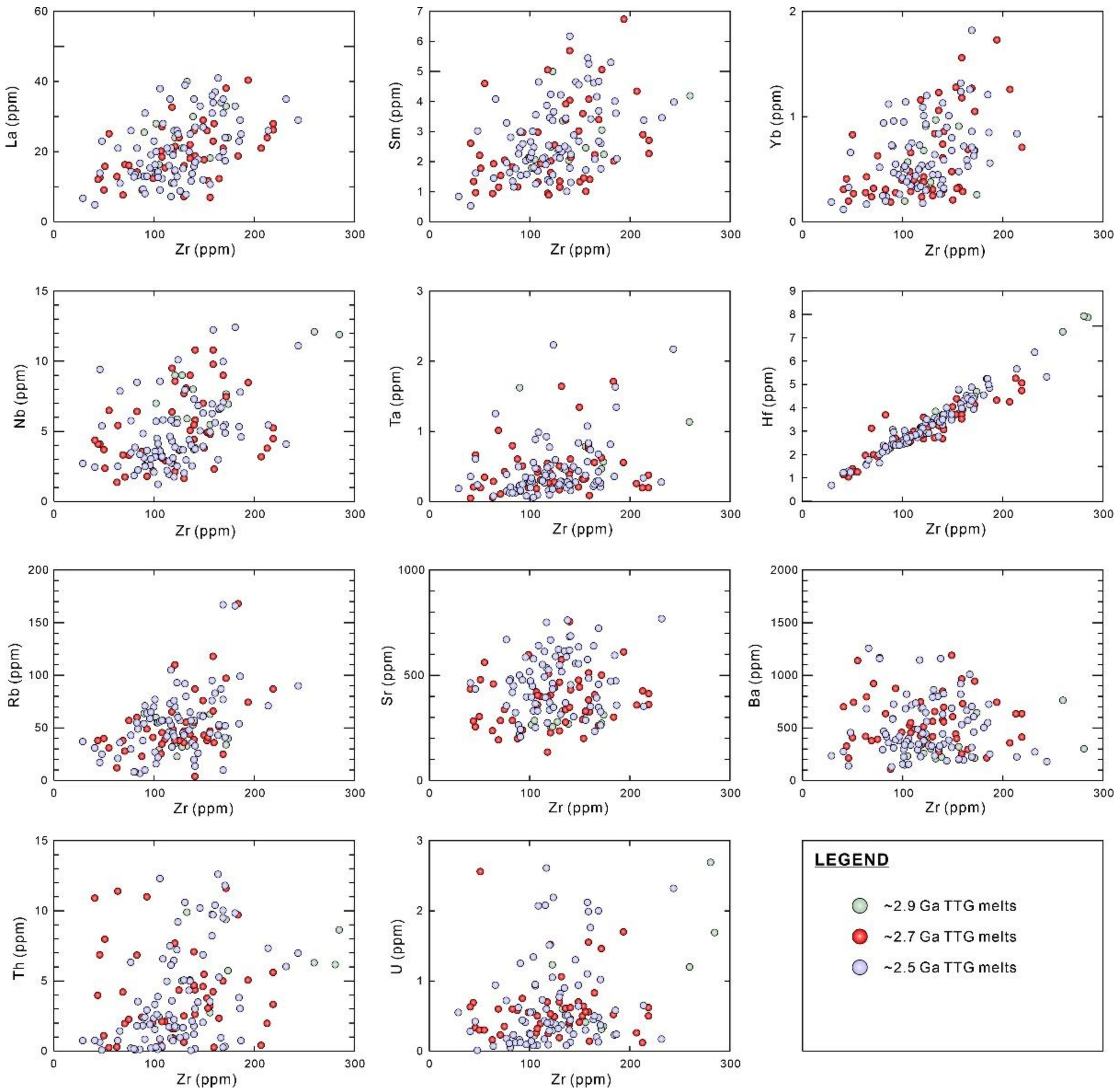

Supplementary Figure 10. Binary element variation diagrams of the crustal-derived Archean TTG melts in the EB of NCC.

Supplementary References

1. Liu, D. Y., Nutman, A. P., Compston, W., Wu, J. S. & Shen, Q. H. Remnants of ≥3800 Ma crust in the Chinese part of the Sino-Korean craton. *Geology* **20**, 339–342 (1992).
2. Zhai, M. G. & Santosh, M. The Early Precambrian odyssey of the North China Craton: a synoptic overview. *Gondwana Res.* **20**, 6–25 (2011).
3. Zhao, G. C., Sun, M., Wilde, S. A. & Li, S. Z. Late Archean to Paleoproterozoic evolution of the North China Craton: key issues revisited. *Precambr. Res.* **136**, 177–202 (2005).
4. Wan, Y. S. et al. ~2.7 Ga juvenile crust formation in the North China Craton (Taishan–Xintai area, western Shandong Province): further evidence of an understated event from U–Pb dating and Hf isotopic composition of zircon. *Precambr. Res.* **186**, 169–180 (2011).
5. Liu, S. J. et al. Neoarchean to Paleoproterozoic high-pressure mafic granulite from the Jiaodong Terrain, North China Craton: Petrology, zircon age determination and geological implications. *Gondwana Res.* **28**, 493–508 (2015).
6. Guo, B., Liu, S., Zhang, J., & Yan, M. Zircon U–Pb–Hf isotope systematics and geochemistry of Helong granite-greenstone belt in Southern Jilin Province, China: Implications for Neoarchean crustal evolution of the northeastern margin of North China Craton. *Precambr. Res.* **271**, 254–277 (2015).
7. Wang, M. J. et al. Petrogenesis and tectonic implications of the Neoarchean North Liaoning tonalitic-trondhjemitic gneisses of the North China Craton, North China.

J. Asian Earth Sci. **131**, 12–39 (2016).

8. Diwu, C. R., Sun, Y., Guo, A. L., Wang, H. L. & Liu, X. M. Crustal growth in the North China Craton at ~2.5 Ga: Evidence from in situ zircon U–Pb ages, Hf isotopes and whole-rock geochemistry of the Dengfeng complex. *Gondwana Res.* **20**, 149–170 (2011).
9. Huang, X. L., Niu, Y. L., Xu, Y. G., Yang, Q. J. & Zhong, J. W. Geochemistry of TTG and TTG-like gneisses from Lushan-Taihua complex in the southern North China Craton: Implications for late Archean crustal accretion. *Precambr. Res.* **182**, 43–56 (2010).
10. He, Y. H., Zhao, G. C., Sun, M. & Wilde, S. A. Geochemistry, isotope systematics and petrogenesis of the volcanic rocks in the Zhongtiao Mountain: An alternative interpretation for the evolution of the southern margin of the North China Craton. *Lithos* **102**, 158–178 (2008).
11. Hou, K. J., Ma, X. D., Li, Y. H., Liu, F. & Han, D. Genesis of Huoqiu banded iron formation (BIF), southeastern North China Craton, constraints from geochemical and Hf–O–S isotopic characteristics. *J. Geochem. Explor.* **197**, 60–69 (2019).
12. Fu, J. H., Liu, S. W., Zhang, B., Guo, R. R. & Wang, M. J. A Neoarchean K-rich granitoid belt in the northern North China Craton. *Precambr. Res.* **328**, 193–216 (2019).
13. van Achterbergh, E., Ryan, C., Jackson, S. & Griffin, W. L. In: Sylvester, P. (Ed.), Appendix 3 Data Reduction Software for LA-ICP-MS in "Laser-Ablation-ICPMS in the Earth Sciences": Mineralogical Association of Canada Short Course **29**, 239–

243 (2001).

14. Anderson, T. Correlation of common lead in U–Pb analyses that do not report ^{204}Pb. *Chem. Geol.* **192**, 59–79 (2002).
15. Ludwig, K. R. Berkeley Geochronology Center Special Publication, vol. 4 (2003).
16. Yuan, H. L. et al. Accurate U–Pb age and trace element determinations of zircon by laser ablation inductively coupled plasma-mass spectrometry. *Geostand. Geoanal. Res.* **28**, 353–370 (2004).
17. Hu, Z. C. et al. Improved in situ Hf isotope ratio analysis of zircon using newly designed X skimmer cone and jet sample cone in combination with the addition of nitrogen by laser ablation multiple collector ICP-MS. *J. Anal. Atom. Spectrom.* **27**, 1391–1399 (2012).
18. Polat, A., Hofmann, A. W. & Rosing, M. T. Boninite-like volcanic rocks in the 3.7–3.8 Ga Isua greenstone belt, West Greenland: geochemical evidence for intra-oceanic subduction zone processes in the early Earth. *Chem. Geol.* **184**, 231–254 (2002).
19. Polat, A. & Hofmann, A. W. Alteration and geochemical patterns in the 3.7–3.8 Ga Isua greenstone belt, West Greenland. *Precambr. Res.* **126**, 197–218 (2003).
