# Supplementary material for: Thermal state and evolving geodynamic regimes of the Meso- to Neoarchean North China Craton
Source: Nat Commun. 2021 Jun 23;12:3888. doi: 10.1038/s41467-021-24139-z (PMC8222299; doi:10.1038/s41467-021-24139-z)
Supplement: Supplementary file 3 — Description of Additional Supplementary Files [file 41467_2021_24139_MOESM3_ESM.pdf]

Description of Additional Supplementary Files

File Name: Supplementary Data 1
Description: The melt compositions of the partial melting experiments on low-K basalts, high-K basalts, tonalites, metagreywackes, metapelites and peridotites.

File Name: Supplementary Data 2
Description: Summary of zircon Lu-Hf isotopic data for Meso- to Neoarchean TTG gneisses from the Eastern Block of NCC.

File Name: Supplementary Data 3
Description: Summary of zircon age data and sampling locations for Meso- to Neoarchean TTG gneisses in the Eastern Block of NCC.

File Name: Supplementary Data 4
Description: Analytical results of major (wt.%) and trace (ppm) elements and calculated parameters for Meso- to Neoarchean crustal-derived TTG gneisses from the Eastern Block of the NCC.

File Name: Supplementary Data 5
Description: Analyzed zircon U–Pb isotopic data and calculated apparent ages for representative TTG gneisses from the Eastern Block of the NCC.

File Name: Supplementary Data 6
Description: Average composition of potential source mafic rocks for Meso- to Neoarchean crustal-derived TTGs compiled from the GEOROC databases.

File Name: Supplementary Data 7
Description: Thermodynamic and trace element modelling of partial melting of Meso- to Neoarchean low-K mafic rocks.

File Name: Supplementary Data 8
Description: Mineral partition coefficients used in trace element modelling of partial melting of mafic rocks (after Bédard 2006 and Martin et al., 2014).

File Name: Supplementary Data 9
Description: List of the parameters and of their values used in the geothermal models.
