# Supplementary material for: Thermal state and evolving geodynamic regimes of the Meso- to Neoarchean North China Craton
Source: Nat Commun. 2021 Jun 23;12:3888. doi: 10.1038/s41467-021-24139-z (PMC8222299; doi:10.1038/s41467-021-24139-z)
Supplement: Supplementary file 4 — Supplementary Code 1 [file 41467_2021_24139_MOESM4_ESM.pdf]

Supplementary Code 1

MATLAB

Build of geotherm model

```
Load initial parameters qT, z, TT, k, A, Δz;
Then,
    a= [];
        for iteration =1:(z*10)
            TB = TT+((qT/k) *Δz) -(A*(Δz^2)/(2*k));
            qB = qT-A*Δz;
            TT=TB;
            qT=qB;
            a(iteration)=TB;
            end
    b= [];
    H=0;
        for iteration =1:(z*10)
            H=H+0.1;
            b(iteration)=H;
            end
Output,
    a, b, figure
```
